# Experiences of parents and caretakers going through the consent process to perform minimally invasive tissue sampling (MITS) on their deceased children in Quelimane, Mozambique: A qualitative study

**DOI:** 10.1371/journal.pone.0286785

**Published:** 2023-06-09

**Authors:** Amilcar Magaço, Maria Maixenchs, Yury Macete, Nelson Escritório, Raquel Mucor, António Calia, António Sitoe, Elisio Xirinda, Pio Vitorino, Mischka Garel, Robert F. Breiman, Agbessi Amouzou, Quique Bassat, Inácio Mandomando, John Blevins, Khátia Munguambe

**Affiliations:** 1 Centro de Investigação em Saúde de Manhiça (CISM), Maputo, Mozambique; 2 ISGlobal, Hospital Clínic—Universitat de Barcelona, Barcelona, Spain; 3 Emory Global Health Institute, Atlanta, GA, United States of America; 4 Department of Global Health, Rollins School of Public Health, Emory University, Atlanta, GA, United States of America; 5 Johns Hopkins Bloomberg School of Public Health, Baltimore, Maryland; 6 ICREA, Pg. Lluís Companys, Barcelona, Spain; 7 Pediatrics Department, Hospital Sant Joan de Déu, Universitat de Barcelona, Esplugues, Barcelona, Spain; 8 Consorcio de Investigación Biomédica en Red de Epidemiología y Salud Pública (CIBERESP), Madrid, Spain; 9 Instituto Nacional de Saúde (INS), Ministério da Saúde, Maputo, Mozambique; 10 Faculdade de Medicina, Universidade Eduardo Mondlane, Maputo, Mozambique; Universitat Luzern, SWITZERLAND

## Abstract

**Background:**

In Mozambique, the Countrywide Mortality Surveillance for Action (COMSA) Program implemented a child mortality surveillance to strengthen vital events registration (pregnancies, births, and deaths) and investigate causes of death using verbal autopsies. In Quelimane district, in addition to the abovementioned cause of death determination approaches, minimally invasive tissue sampling (MITS) was performed on deceased children <5years of age. This study focused on understanding deceased children parents’ and caretakers’ experiences of the consent process to perform MITS in order to contribute to the improvement of approaches to cause of death investigation and inform efforts to maximize acceptability of mortality surveillance activities.

**Methods:**

A qualitative study was conducted in six urban and semi-urban communities in Quelimane district. A total of 40 semi-structured interviews with family members of deceased children and 50 non-participant observations of the consent process were conducted to explore their experience with informed consent request to perform MITS on their child. Data analysis of the interviews and observations was thematic, being initially deductive (predetermined codes) followed by the generation of new codes according to the data (inductive).The Consolidated criteria for reporting qualitative research (COREQ) guidelines for reporting qualitative studies were performed.

**Findings:**

Although most participants consented to the performance of MITS on their deceased child, some stated they had not fully understood the MITS procedure despite the informed consent process due to unclear information and their state of mind after their loss. Consenting to MITS and doing so with family members disagreeing were also identified as stress-enhancing factors. Participants also described dissatisfaction of family members, resulting from the condition of the body delivered after tissue collection. In addition, the waiting time to receive the body and resulting delays for the funeral were considered additional factors that may increase stress and compromise the acceptability of MITS.

**Conclusion:**

Family experiences were influenced by operational and logistical issues linked to the procedure itself and by it being in tension with social and cultural issues, which caused stress and discontentment on parents and caretakers of deceased children. The main factors that contributed to the experience of going through the MITS process were the state of mind after the death, complex decision making processes within the family, washing of the body for purification after MITS and seepage, and limited understanding of consent for MITS. When requesting consent for MITS, emphasis should be placed on transmitting clear and understandable information about MITS procedures to participants.

## Introduction

Despite significant reductions over the past two decades, an estimated 5.2 million children die every year before reaching five years of age, with the vast majority of those deaths disproportionately clustering in low-income countries in sub-Saharan Africa. In this region 1 in 13 children die before the age of five years, a figure 16 times higher than the average ratio of 1 in 199 in high-income countries [1]. In Mozambique, the under-five mortality rate fell gradually from 170 deaths per 1,000 live births in 2000 to 71 deaths per 1,000 live births in 2020 [2], meaning that one of every fourteen babies do not survive to their fifth birthday. Most resource-constrained settings lack accurate and acceptably methods for cause of death (CoD) determination leading to a poor understanding of global child mortality and health. Less than 20% of the 192 countries in the world have high-quality death registry data, and more than 30% do not have any specific mortality registry data [3]. For this reason, improvements in tracking the mortality of children under five are at the forefront of public health priorities globally. Mozambique faces the paradoxical challenge of a high child mortality rate coupled with low coverage of vital events records [4–6]. Since 2018, the Countrywide Mortality Surveillance for Action (COMSA) program is being implemented to collect data on vital events in Mozambique while investigating the causes of death (CoD) in children under 5 years through verbal autopsies [3,7]. Acknowledging that verbal autopsies are subject to a certain degree of inconsistencies and diagnostic errors at an individual level, COMSA uses data on Minimally Invasive Tissue Sample (MITS) in deaths among children under-five (except for those caused by trauma and other accidents such as drowning and burns), in order to complement and calibrate the information derived from verbal autopsies, having selected Quelimane district in Zambézia province as the site for this study [6,8,9]. Of note, this a setting were cause of death determination does not occur routinely due to lack of equipment and expertise. Hence, MITS do bring additional value to cause of death determination.

As MITS is a procedure that allows the extraction of tissue and body fluid samples from a set of key organs without the need to open the body. It is, therefore, believed to be more acceptable than the complete diagnostic autopsy (CDA) [6,9]. MITS implementation in Mozambique, as well as in other LMICs, has been shown to be feasible [6,9,10]. Two key components of MITS feasibility are people’s perceptions of MITS, which is seen as a technique that minimally disfigures the body of the deceased, resulting in a milder negative emotional impact on families, and the relatively high acceptability in the majority of places where it has been implemented [11–13].

While the aforementioned studies have suggested that the MITS approach is feasible and acceptable in settings where other more disfiguring postmortem procedures are complex to conduct and poorly accepted, the measurement of acceptability has been limited to the consent process, and not much has been explored beyond the act of consenting to MITS. Therefore, we set out to explore the dimensions of acceptability expressed through the experiences of those who have gone through the process of consenting to MITS on their deceased relatives in specific contexts. Such studies are needed in settings where the investigation of CoD through minimally invasive procedures is ongoing or is being considered, and are of particular relevance in places like Quelimane District, where a history of myths, rumours and negative perceptions on specific public health initiatives and interventions is known to exist [14]. This study aimed to understand the experiences of parents and caretakers of deceased children about the consent procedure for MITS, including the decision-making process for MITS, in order to contribute to improved approaches to cause of death investigation and inform efforts to maximize the acceptability of mortality surveillance activities.

## Methods

### Study site and population

This study was conducted in Quelimane district, the capital of Zambézia province, one of the provinces with the poorest health indicators in the country [15]. Administratively, Quelimane is a district covering an area of 117 Km^2^, with 349,842 inhabitants. It is located by the Bons Sinais River, and about 20 Km from the Indian Ocean. The district has urban and rural areas. The urban area comprises the City of Quelimane–the capital of Zambézia province, with 71.7% of the total population within the district [16]. The population is mostly of *Chuabo* ethnicity, the dominant ethnic group, and Christianity is the main religion (60.2%), with nearly a fifth of the population (18.9%) identifying as Muslim [16]. Quelimane district incorporates a municipality, administered by an elected local government, which covers 5 Urban and one rural Administrative Posts. Quelimane hosts 21 health facilities, including 17 health centers, two peripheral health posts, a general hospital and a tertiary level central hospital–the Central Hospital of Quelimane (HCQ), where the minimally invasive tissue sample procedure was introduced in March of 2019 in the context of the COMSA program in partnership with the Child Health and Mortality Prevention Surveillance (CHAMPS) network. CHAMPS was established to collect standardized, population-based, longitudinal data to determine with greater specificity the causes of under-5 mortality and stillbirths in sub-Saharan Africa and south Asia, and to improve the accuracy of cause of death ascertainment [17]. Additionally, community entry and engagement procedures were put in place to raise awareness about COMSA and MITS, consisting of community workshops also known as Participatory Inquiry Into Community Knowledge of Child Health and Mortality Prevention (PICK CHAMP) [18] followed by mapping and identifying key stakeholders, mortality surveillance outreach meetings, sensitization for MITS acceptance and other mortality surveillance activities.

### Study design

A qualitative study was conducted as part of the second phase of formative research to inform the COMSA program, with particular attention on experiences and key challenges during MITS implementation in Quelimane. The first phase of COMSA formative research, which was conducted in October 2018 –prior to the introduction of MITS in Quelimane -, aimed to understand local attitudes and perceptions regarding the death, postmortem body care as well as potential barriers and facilitators to the implementation of MITS.

Following ethnographic and phenomenological approaches [19–21], this component of the study sought to register the experiences and meanings of families of deceased children during and after consenting to MITS on their deceased children’s bodies. The study focused on participants’ views, behavior, and attitudes around the MITS procedure; interactions with family members for decision making for acceptance (or not) of the MITS procedure, and their interactions with the COMSA consent team and other factors and events related to MITS.

### Study participants and recruitment

This study, targeted parents and caretakers of deceased children after confirming all the eligibility criteria for MITS namely non accidental deaths occurring in stillborn and Children aged 0–5 years who are residents of the designated surveillance area reported within 24 hours if kept at room temperature and 36 hours if kept refrigerated and if parents provided a written consent to MITS procedure.

Throughout the second phase of the COMSA formative research (from April to July 2019), 80 child death cases that were eligible for MITS were identified; parents or caretakers of these deceased children were approached by the clinical study staff, who requested consent for the performance of MITS. The informed consent process took place at the pediatric, maternity, and obstetrics wards as well as the nursery of the HCQ. Fifty of these informed consent procedures were randomly selected to be observed by the Social and Behavioral Science (SBS) team members, who, based on these observations, identified potential interview participants. Regardless of accepting or refusing the performance of MITS, the clinical study staff held short conversations with the identified parents or caretakers to explore the possibility to participate in an interview about their experience, to be conducted up to 4–6 weeks later. Although 50 were considered for interview, during the process of confirming availability for interviews only 40 participants were successfully contacted and interviewed. With their permission, some of the participants were sought and interviewed at their homes, and others were approached and located via a phone call when they could not be reached at the address provided at the time of recruitment. The remaining 10 potential participants were not accessible, and therefore considered not included in the study for the following reasons: refusal and unwillingness to participate in the interview, loss to follow-up due to wrong address, and unavailable (offline) phone numbers.

### Data collection procedures

A combination of two qualitative data collection techniques, non-participant observations and semi-structured interviews (SSI), were-conducted. Non-participant observations were carried out during the informed consent request from parents and caretakers of deceased children and while they were waiting for the death certificate or the body to be returned to the family after the MITS procedure. This technique generated field notes, oriented by observation guide lines (S1 Appendix), and allowed to capture data on behavior and attitudes of potential participants around MITS, and their interactions with the consenting team of COMSA.

Semi-structured interviews were carried out by SBS team members, in the individuals’ homes or in other places of participants’ chose (i.e. HCQ, in a school near the participants’ homes and at their workplaces). The interviews, which were based on standardized interview guidelines with open-ended questions (S2 Appendix and S3 Appendix), lasted approximately 60 to 90 minutes and covered sociodemographic characteristics, perceptions of MITS implementation, experiences during the consent process and after MITS procedure.

Each data collection strategy (Observation, interview to consenters and interview to non-consenters) followed its own topic guide or script, mostly formed by open-ended questions about views and perceptions about the informed consent process and understanding the information given during consent, decision making for acceptance (or not) of the MITS procedure, and other MITS-related factors and events. Four interviewers, specifically trained for the purposes of the study, and one social scientist, who acted as the overall study coordinator, collected data. The study was overseen by a senior social scientist based in Maputo. Data collection was carried out in Portuguese or in the local language (*Chuabo*), depending on participants’ preference.

### Data management and analysis

All SSI were digitally recorded and fully transcribed by two experienced external transcribers and one internal transcriber from the study team. All of them were fluent in *Chuabo* and Portuguese and received specialized training in transcription techniques based on the study’s standard transcription procedures. The interviews conducted in *Chuabo* were simultaneously translated into Portuguese during transcription.

The transcribed data were coded with support from *NVIVO12*® (QSR International, Inc.–Australia) qualitative analysis software. These data were then read and coded following the themes and thematic categories created and then analyzed based on the phenomenological approach that characterizes the experiences lived by the research participants [22]. The observation field notes produced by social science research assistants were digitalized in MS word and coded in a separate NVivo codebook and then these data coming from the observations and interviews were compared and highlighted important differences and complementarities with each other. Therefore, our analysis was conducted through an exercise of reading summarizing and comparing the information from the codes extracted in NVivo. This procedure, helped to improve the process of analyzing and constructing analytical categories.

In this study we used inductive and deductive coding [23]. For the observations data analysis was purely inductive. For the interviews, it began with a deductive approach based on a predetermined set of constructs, followed by an inductive approach in which the emerging themes themselves from the data could be incorporated into the initial framework. Thus, in a first cycle of coding, the interviews were coded using predetermined themes. Based on themes emerging throughout the coding process, reading of interview transcripts and analysis, a second cycle of coding was applied (Table 1). This data processing allowed for the creation or re-adaption of codes and helped to organize and sort the text of the SSI and non-participant observation field note into thematic categories. The information entered into the thematic categories were then read and interpreted according to the overarching themes related to the study objectives. This thematic analysis focuses on the perception about the informed consent procedure, understanding of the information given during informed consent, the decision-making process for accepting or refusing the MITS and other factors and events related to death and MITS. Thus, the interpreted data were organized and written up into the overarching themes that make up the content of this paper.

**Table 1:** Themes analyzed: The determined and emerging themes within the analyzed narratives.

Predetermined themes	Emerging themes
Perception about consenting to MITS	*Experiences of consent after the death*
	*Perception of susceptibility*
Perception about MITS	*Perception about the benefits of MITS*
	*Who makes the decision to accept/refuse MITS*
	*Perception of the time to conduct MITS*
Decision making to perform MITS	*Reasons for accepting MITS*
	*Reasons for refusing MITS*
General experience	*N/A*
Experience post MITS	*Negative experiences*
	*Translating the bodies of deceased children to the community*
	*Positive experiences*

### Ethical aspects

The project was approved under the CHAMPS protocol, by the National Committee for Bioethics in Health of Mozambique (285/CNBS/16). This qualitative study received ethical approval (CIBS-CISM/013/2018) by the Institutional Committee on Bioethics in Health of the Manhiça Health Research Centre (CIBS-CISM). Administrative authorizations were provided by the Ministry of Health (MoH), the Zambézia Provincial Health Directorate and the Municipal Authority of Quelimane City. Administrative authorization to engage with community members and conduct the study was also first requested from neighborhood secretaries and the heads of each administrative post included in the study. Additional consent to conduct follow-up interview was previously incorporated orally during the consent process for the MITS. During this process, participants were also informed that observers were present and taking notes of the entire process since the child’s death and oral informed consent was provided. Participants were given the opportunity to accept or decline that the information obtained be used for the purpose of this research.

## Results

### Sociodemographic characteristics

Forty (n = 40) Semi-structured interviews were conducted with parents and caretakers of deceased children under five years of age who were recruited to participate in the study. The interviewees’ sociodemographic characteristics are summarized in Table 2. Of the total interviewees, 36 were parents of deceased children, and 4 caregivers and relatives consisting of grandparents or uncles/aunt of the deceased children. The median age of the respondents was 24.5 (Range, 17–38), 77% were female, and 40% had no formal education. Among the respondents, 37% were workers in the informal sector consisting of Bicycle Taxi Drivers, Domestic Workers, bricklayers and small-scale traders; 55% were mothers of the deceased children and 32% were fathers and the remaining participants were other relatives such as uncles, aunts and grandparents.

**Table 2:** Socio-demographic characteristics SSI participants.

	Accepted MITS n = 37 (92%)	Refused and Loss of follow-up for MITS n = 3 (8%)	Total n = 40 (100%)
**Median age (range)**	24 (17–38)	25 (18–31)	**24,5 (17–38)**
**Gender**			
**Male**	8	1	**9 (23%)**
**Female**	29	2	**31 (77%)**
**Education Level**			
*No formal education*	16	0	**16 (40%)**
*Primary education*	7	1	**8 (20%)**
*Secondary/higher*	12	2	**14 (35%)**
*Professional Technician*	2	0	**2 (5%)**
**Marital status**			
*Never married*	15	1	**16 (40%)**
*Married or cohabiting*	12	2	**14 (35%)**
**Widow**	10	0	**10 (25%)**
**Occupation**			
*Formal Worker*	2	1	**3 (8%)**
*Informal Worker/business*	13	2	**15 (37%)**
*Student/No job*	22	0	**22 (55%)**
**Relation to the child**			
**Father of the deceased child**	12	1	**13 (32%)**
**Mother of the deceased child**	20	2	**22 (55%)**
**Maternal-Grandmother of the deceased child**	3	0	**3 (7%)**
**Maternal Uncle of the Deceased Child**	1	0	**1 (3%)**
**Maternal aunt of the Deceased Child**	1	0	**1 (3%)**
**Location**			
**Rural**	12	1	**13 (32%)**
**Urban**	25	2	**27 (68%)**

### Experiencing the MITS consent process

Shortly after the announcement of the child’s death at the HCQ, contact was made to parents and caretakers of deceased children leading to the process of requesting consent for MITS procedure. In this process, 80 parents and caretakers of children who died either in the pediatric, nursery or maternity wards were approached for MITS consent. Of these, 50 underwent the full process of non-participant observation of the MITS informed consent process. Table 3 below illustrates the details of the description of the consent data.

**Table 3:** Numerical description of consent data for MITS.

Indicators	Observed n = 52 (65%)	Unobserved n = 28 (35%)	Total n = 80 (100%)
**Approached for MITS**			
** Consented for MITS**	50	28	78 (97.5%)
** Refused MITS**	2	0	2(2.5%)
**Performed MITS**			
** Yes**	50	27	77(96.5%)
** No**	2	0	2(2.5%)
** Loss of follow-up for MITS**	0	1	1(1%)

The process of requesting consent to perform MITS begins with the notification of the death managed by the healthcare provider assigned to the sector where the death happened to the COMSA call centre (Death notification call centre in the context of mortality surveillance). During the time this research was conducted, two people constituted the consent team, and they worked in shifts as permanent staff of HCQ. For each MITS-qualifying death, the Call Center called the consenter who went to the sector or ward to confirm the information and initiate the request for consent to the father, mother or any eligible relative of the deceased child available for consent. During this process, MITS consenters explained to the deceased child’s family member the specifics regarding the MITS procedure, how it is performed, its purpose, the time it takes and the need to provide consent to perform the MITS procedure. Most of the observed participants consented to perform MITS on their children’s bodies.

Interviews revealed that some participants did not expect they would be approached to provide consent for MITS or any postmortem procedure in the hospital because they were unaware of the implementation of MITS in HCQ. These participants mentioned that after the explanation of the objectives of the MITS procedure, they had difficulty in simply either accepting or refusing, mainly because they were in shock after receiving the news of their child’s death and also because MITS was to them an unfamiliar procedure to be carried out on a recently deceased child.

*“At that moment*, *it was difficult for us to say anything*. *My wife was still hospitalized*, *and I didn’t know what to do”* (Father of a deceased child, accepted).

The observation team confirmed that many parents and caretakers were surprised by the information about MITS and the request for consent to carry it out. And at this moment, we observed and took notes of their comments and questions of concern after they had consented to MITS:

*“Will this MITS take long*? *How long will this take now*? *After that*, *will we know what kills our child*?*”* (Observation Notes, Various).

On the one hand, data from the observations revealed that the consent to perform MITS was easily provided when the parents or caretakers of the deceased child already knew, through community engagement activities, about the COMSA program and the implementation of MITS in the HCQ. Some of the SSI participants who consented to MITS declared that even before being approached by the consent team, they had already expressed their desire to know the causes of death of their child through MITS.

*“When that death occurred*, *we were already wanting to know what had happened for our child to die*, *and when that lady* [consenter] *came*, *I immediately accepted because I had also seen the campaign about that program (COMSA) in the neighborhood*.*”* (Mother of a deceased child, accepted).

On the other hand, prior knowledge about COMSA and MITS did not necessarily ensure the acceptance of MITS. One of the participants who identified himself as a healthcare professional refused instantly to give consent for MITS to be performed on his recently deceased son. This participant mentioned that although he was familiar with the COMSA program and its approach, he was not prepared to accept having the MITS procedure performed on his child’s body.

*“It was difficult for me to accept because*, *in fact*, *what happens is that we are not used to doing such a thing after a person dies… and for our relatives who don’t even know your project*, *for them*, *it would be more difficult to accept”* (Father of a deceased child, Refused).

Some participants who consented to MITS showed reservations and hesitation because of concerns related to Islamic religious beliefs. These participants explained that although they understood the information, they needed to ensure that the procedure and the time for its implementation would not conflict with their religious beliefs and practices related to funerals and burial.

*“Our family is Muslim and we have our rules that we can’t break”* (Father of a deceased child, accepted).

### Decision making process to accept MITS

Several decision-making challenges were observed during the consent process for performing MITS. Decision making to provide consent for MITS varied among participants. In some cases, the decision-making process involved a group of family members of the deceased child (such as uncles, grandparents and other family members who were supporting the parents at the hospital), and in other cases, it only involved the parents.

In situations where the decision to accept or refuse MITS was made solely by the child’s parents, acceptance of consent to MITS was easily provided. In these cases, the parents’ decision to accept MITS depended on how they assimilated the information about their child’s death.

However, consent processes only involving parents were not always straight forward. One of the interviewed participants who did not accept MITS justified having refused because, first, he did not agree with the performance of the procedure without the approval of his wife, who was currently hospitalized, and second, because the decision for any postmortem procedure did not depend exclusively on them but also on other family members who were not present at the moment of the consent process for MITS.

*“I can’t accept it alone*, *the owner* [mother of the deceased child] [hospitalized] *was dealing with labor pains*, *so I couldn’t accept it*, *I can’t accept it without her knowing”* (Father of a deceased child, Refused).

In cases where decision-making involved other family members advising and being consulted by the parents, the process for providing consent was complex and time-consuming. In these cases, they only decided on whether or not to perform MITS after consulting other, usually absent, family members, who according to the participants, were considered the decision makers in the family. These family members were often the child’s grandparents, uncles and aunts and uncles closest to the child’s parents’ family. One of the participants mentioned that not consulting their elders could be considered disrespectful and could lead to family problems based on accusations and blame.

*“We have our elders who must be consulted for everything*. *A decision like this they must be consulted*, *when we don’t consult them they can raise big problems in the family*, *if anything goes wrong they will make it your fault”* (Father of a deceased child, accepted).

Participants mentioned that in sensitive situations such as the moment of death, decisions about which steps to follow including any postmortem procedures if needed, are always fraught with demands from family decision-makers, especially when it is a difficult decision as one of the participants mentioned: *"Because it is a difficult decision*, *we always have to consult our elders*, *those who stayed at home waiting for us"* (Mother of a deceased child, accepted). For the participants, this situation worsens in scenarios where those approached by the consent team do not know if the manipulation of the body after death is aligned with their traditional and religious beliefs. Often, this is the reason why decision makers in families are consulted. In addition, there is also the fear of the reaction of other family members when they learn that the performance of MITS in the child’s body has been accepted.

"*You can’t override the decision of those who are older*, *we are ’children’ to decide this for ourselves*" (Mother of a deceased child, Refused).

Despite the complexities of involving different segments of the family in the decision-making process, participants reported a positive experience when decisions were made jointly by the family of the child’s maternal and paternal family members, because it reduced discontent and tension within the family. One of the participants also pointed out that:

*"When they all want it*, *we don’t have any problems*, *because it is important that the whole family wants to know the causes of the child’s death"* (Mother of a deceased child, Accepted).

After accepting consent, some parents expressed concern about the (un)certainty whether they had made the right decision in accepting consent to perform MITS, because they feared reprisals from their elders if MITS failed to bring them relevant information and solutions for the disease that killed the child.

### Comprehending the MITS procedure

Most parents and caretakers who participated in the consent process mentioned that they were informed about the procedure that would be done on the body of their deceased child. After the explanation, some of them were enthused by the importance of MITS and the idea of knowing the causes of death of their children. According to these participants, the MITS procedure would help prevent the deaths of other children yet to be born. Though for them, the information generated on the causes of death of the children subject to MITS would facilitate the care and future approaches to improve the children’s health.

*“We do understand the benefit of this [MITS]*, *it has advantages for our other children”* (Father of a deceased child, Accepted).

Although many more cases of accepted consent were reported compared to refusals, there were different understandings of usefulness of the MITS procedure, even among those who accepted MITS. While some participants considered MITS to be a beneficial innovative procedure, others viewed it as yet another biomedical intervention that still needed to be understood. Likewise, several concerns that may influence the future acceptability of MITS were identified: apparent skepticism towards the procedures performed in the MITS room and the negative experiences after the performance of MITS, such as the waiting time of the body for the funeral ceremonies and the bleeding of the body at the time of the funeral.

Regarding skepticism towards MITS procedures, the observations revealed that the dynamics of managing the moments of grief and pain of loss reduced the ability of parents and caretakers to understand the explanations of the MITS procedure. This limitation in understanding the explanations describing the MITS procedure contributed to doubts and hesitation among some family members and delays in the consent process.

According to some of the parents and caretakers who consented to MITS and received the body after the MITS procedure, several doubts arose about the procedures performed in the MITS room. In addition, there were questions about how or when the results of testing and cause of death information would be delivered, despite the fact that these issues were explained during the informed consent process. In addition, some of the family members who consented to MITS mentioned that not being invited to watch the MITS procedure made unaware of what exact procedures were performed in the sample collection room.

*"*…*first that nurse said I could watch*, *but then when they went in there [MITS room]*, *they didn’t call us to come in and watch"* (Uncle of a deceased child, Accepted).

In these cases, only two participants mentioned having asked for additional explanation on how the whole procedure would take place and when the results would be delivered. One of them said:

*"Our family members who were there at the hospital that day kept asking a lot of questions*… *they wanted to know about a lot of things*, *because of those things* they [The consenters] *talked about they didn’t understand at all"* (Father of a deceased child, Accepted).

In addition, misunderstandings about MITS and the time required to perform them resulted in some families not claiming the bodies of their deceased children after the execution of MITS because they had thought that the bodies would be delivered to their homes for burial.

*"There they said it was to leave it* [the body], *right there to do this MICS thing* [MITS], *but then they didn’t bring us* [the body] *until now [*…*] were we supposed to pick up* [the body] *at the hospital*?*"* (Aunt of a deceased child, Accepted).

*"We were left there without knowing what we are going to do*…*if our child stays* [At the hospital, after MITS] *or if we can take it"* (Mother of a deceased child, Accepted).

A father of a deceased child who consented to MITS described his experience in that way: *"it was a sad situation because we don’t know where the child’s body was left*…*we waited a long time to receive the body to perform our funeral ceremonies"* (Father of a deceased child, Accepted).

### Experiences after the MITS procedure

Although some families chose to leave the hospital when they consented, several who remained at the hospital described the waiting time as unpleasant and difficult to manage. Waiting caused anxiety and nervousness, which in extreme cases culminated in disgruntled family members speaking out loud and retaliating.

*"*…*When you stand there* [at the hospital] *waiting*, *after your child dies*, *you get more nervous*, *tired*, *you just want to go home"* (Father of a deceased child, Accepted).

Anxiety not only came from the length of time it took to complete a MITS but also from the hurry to return home, in turn, influenced by pressure from other family members who were also waiting for the body to arrive home for the funeral ceremonies to begin.

*"Even I who was in the hospital*, *even those uncles who were there at home*, *everyone was already complaining that it was taking so long for us to take the child home"* (Uncle of deceased child, Accepted).

The waiting time for the body to be released (About 2 hours and 30 minutes), for the funeral and the bleeding of the bodies were mentioned by the participants. Some participants described that after having experienced the difficulty of time management during the execution of MITS, when they returned to the community, at the time of body washing for the funeral ceremony, they found blood dripping from the body. These participants described that seeing the blood dripping at that moment of grief was a shock to all the family members who participated in the process of body preparation for burial. During the interview, a father of one of the deceased children expressed irritation about the bleeding, and mentioned that he sought out the CHAMPS staff to ask for clarification about this bleeding.

*"*…*When we saw that blood coming out*, *every family member was scared and angry because normally we don’t accept that procedure (MITS)*, *we only accepted it to know what killed the baby" (*Father of deceased child, Accepted*)*.

According to the participants, several members of their families were concerned about the seepage during the funeral and its relationship with the procedures performed on the body of the deceased child. One of the participants, mentioned that they did not understand how the body was delivered while bleeding after undergoing an intervention that was described as minimally invasive.

*"…they said they weren’t going to open the body*, *then we’re seeing blood*, *how*? *When they explained they didn’t say that blood was going to come out"* (Mother of a deceased child, Accepted).

The maternal uncle of one of the children, mentioned that when he came across the seepage he immediately imagined that something wrong was done on the body. Now this uncle described the scenario with a tone of distrust and uncertainty, as he did not know that after the child’s death, the body had gone through a sampling procedure at the hospital, having been informed after questioning about the bleeding.

*"That situation couldn’t happen like that*…*we’re here at home*, *we’re seeing blood coming out from the child body*, *something was done to our child*, *something they don’t want to explain well"* (Uncle of deceased child, Accepted).

## Discussion

In this study we explored the experiences and feelings of parents and caretakers of deceased children undergoing the MITS procedure and consent in the context of a health surveillance and prevention of infant mortality program in Quelimane. The results of this study suggest that the state of mind after the loss of a beloved one, the process of requesting consent and explaining about MITS, understanding the MITS procedure, the dynamics of decision making in the family, the waiting time for the return of the body and associated potential interference with burial arrangements, and the condition of the body after MITS were the main dimensions of the experiences of parents and caretakers during all procedures involving consent to and performance of MITS.

Our results highlight that although most of the participants included in this study consented for MITS, matching the same overall MITS acceptability and performed rate (96.5%) among approached participants recruited for MITS in the same period, some showed reservations and hesitation, both prior to and after consenting to MITS, due to a number of concerns, including learning about MITS for the first time almost immediately after learning about the death of their child. During this process, state of mind of the parents was a major barrier to a straight forward consent process, followed by the complex decision-making dynamics among families. Nevertheless, during the implementation period of this study CHAMPS recorded low MITS refusal rates from only two parents of deceased children and a loss to follow-up of one person who accepted consent for MITS and later left the hospital taking the body of the deceased child with him prior to the MITS performance, without informing the MITS team.

The results of the study showed that in some cases the acceptability of MITS was limited to a complex decision-making process which often varied from family to family, as some decisions depended on family members other than the child’s parents. This finding is similar to experiences reported in a study in Northern India where the parents of the deceased child did not have the authority to decide whether to accept or refuse to perform a postmortem procedure on the child [24]. In other studies conducted in low-income countries in Asia and Africa, the decision-making process at sensitive times such as death seems not limited only to the parents or caretakers who took care of the child before they died, but is a process that involves all family members, especially the oldest and most experienced in the family, those having the decision making power over almost everything in the family [11,25,26]. Furthermore, families found themselves in the paradoxical situation whereby precisely those considered to be the decision-makers of the postmortem events remained at home waiting for information coming from the hospital and for the return of the body home for the ceremonies, while the non-decision makers were at the hospital being requested to consent to MITS. This triggered anxiety and desire to return home influenced both by their lack of autonomy to consent to MITS and by the pressure made by those family members who remained at home, considering that those family members who stayed at home are the legitimate decision makers of the postmortem events. As mentioned in a study by Munguambe et al. [26], in cases of death of young children (stillborn and babies) grandparents take the lead in traditional postmortem events, completely sparing the mother from any decision-making role.

Some parents hesitated to consent because of uncertainties around whether traditional and religious practices allowed or prohibited postmortem procedures. In this regard, there was unwillingness to make a decision unless key decision makers in the family were present or consulted. Relatedly, there was the concern about being negatively judged by other family members for having made the decision to accept or refuse the MITS procedure; there was a concern about making a decision against the principles or rules of their communities, as described in a previous multicenter socio-behavioral study [11]. Furthermore, in settings where neonatal mortality accounts for a major portion of under-5 mortalities, a hospital infant death is likely to occur alongside maternal morbidity or mortality [27], leaving families in a more limited position to process sensitive requests such as a postmortem examination in the absence of the mother. This may also suggest that there is a need to massify community awareness efforts about MITS currently being conducted in the HCQ, in order to minimize the surprise factor.

Undergoing the consenting process with the associated failure to understand the explanation of the MITS procedure influenced skepticism toward MITS procedures. These doubts can culminate in perpetuation and spread of (mis)information or rumors about the MITS procedure [28,29].

Uncertainty regarding the procedures performed and the samples extracted in the sample collection room, coupled with not being invited into the procedure room to observe the MITS, may have created skepticism when it did not previously exist (among family members who were trusting and accepting during the consenting procedure). The procedure though minimally invasive, can still be upsetting for parents or caretakers and may be unpleasant to watch. The failure to understand the procedure and the time frame for performing MITS, as well as instances of not returning the body while the child’s relatives are still in the hospital has resulted in unpleasant experiences for some family members, resulting in their regretting consent to the procedure. This concern was also observed in India and Pakistan where cultural concerns and delays in returning the body for burial were described as common reasons for potential refusals [30]. This was also reviewed among families’ members who had said “yes” to MITS but were later shocked from realizing about certain inconveniences linked to the procedure (timing and bleeding). This made it appear that the participants had given consent unwillingly. This could potentially be detrimental to the MITS study. Therefore, in addition to clear awareness, the consent process for MITS should be revised to anticipate these issues.

In addition, MITS teams are obliged to think about the responsibilities (or shared responsibilities with the families) regarding body release, claiming and transportation, and these should be discussed thoroughly with the families before and during the consent process. Logistical or financial support to families on body transportation back to the community could be an approach addressing this challenge. Although only 3 refusals were contributing to this analysis, these can be capitalized as "case studies" that can help understand further real barriers to acceptability. We also note that bodies left at the hospital at the time of MITS execution, whether by the free will of the family or a misunderstanding, were influenced by time constraints of the families of the deceased child and the desire to return home quickly. Similar results were also found in studies conducted in Pakistan and southern Mozambique, where the desire to return home after death information and the desire to perform funeral ceremonies was urgent and the time factor is a significant concern for child-directed postmortem examinations [26,31]. These data contrast with what was reported in a study carried out in Uganda and Kenya, which found that in situations where a newborn baby dies after the parents have visually confirmed the death, the body is often left behind in the health care facilities [32].

In turn, for the cases where the bodies were taken to the community after the MITS was performed, emotional shock events were reported due to bleeding during the body washing procedure in preparation for the funeral. Although MITS was accepted, the seepage events clash with all the cultural values and traditional or religious practices attached to these families. According to the literature on minimally invasive postmortem procedures, this is mainly aggravated because body fluids were leaving the puncture sites after the MITS procedure and happen due to the short time gap between death and sample collection; the blood was still unfixed and therefore, able to move into the puncture sites when the body was moved [33,34]. Our results suggested the beginning of speculative comments emerging from family members who were unaware of the conduction of MITS on the body that was bleeding. This situation may pose a risk to future acceptability of MITS, and trigger rumors related to MITS procedures [28], especially since the discontent of certain members of the families who accepted MITS was notable.

Importantly, this study reiterates that consenting to MITS is a process whereby there can be fluctuations between being more inclined to say "yes" or to say "no" and that this positioning can vary among the members of the same family, therefore an ultimate and apparent collective "yes" might conceal a few "no’s” on the part of the less vocal family members and vice versa. On the other hand, the inclination to say “yes” or “no” may vary with time and along the process of MITS, as even among family members who say “yes” in the beginning, their views might change during or in the end of the MITS procedure, as they begin to factor in certain elements (such as timing and the adverse impact on the body) that if known to them in advance of the consent, could have altered its outcome. This is not to say that more aware family members would be more likely to say “no” compared to less aware ones, but to encourage the discussion of how to capitalize increased knowledge about MITS among families and communities so that it translates into less tension between family members and less complication and hesitancy in the decision-making process.

The results of the study show that despite the high acceptance of MITS, there were challenges related to negative experiences and episodes of discontent from parents and caretakers of deceased children after the acceptance of consent for MITS and the actual execution of MITS. Our study revealed further dimensions regarding experienced acceptability in that even though this study’s participants were faced with a real scenario of informed consent request to MITS (prompting experienced acceptability) they were faced with different experiences: a quasi-hypothetical one, where MITS is explained to them in a way that requires their abstraction and speculations about risks, benefits and consequences, followed by a real-life scenario (in the waiting and after the release of the body—factual acceptability) where all the elements that influenced their decision were actually experienced. This was contrastingly notable in previous studies, where authors just distinguished between theoretical or hypothetical and actual or experienced acceptability [11,26]. However, in order to make this finding more conclusive, it is recommended that studies also focus on retrospective discussions with participants who are given a chance to share their thoughts about MITS after they have experienced it (post-MITS acceptability), as well as studies with health care providers who interact with parents to obtain consent and conduct the MITS procedure. Thus, more studies of a qualitative nature are needed to understand the experiences and to deconstruct the concept of acceptability by taking into account factors surrounding acceptability. This can help improve and the mortality surveillance implementation in areas such as the district of Quelimane, Mozambique.

Broadly, lessons were learned that go beyond mortality surveillance. First, the importance of a more comprehensive awareness raising approach about new interventions incorporated in existing health system structures. Second, the need to be constantly prepared to address the changes in perception and acceptability of sensitive and complex interventions perceptions evolves with time and experiences. And finally, there is a need not only to anticipate the barriers and facilitators but also to guaranty social and culturally appropriate solutions.

### Strengths and limitations of the study

This qualitative study has notable strengths and some important limitations that are worth highlighting. By the nature of the study and the type of information collected, it presents points that can be used to suggest some changes in the procedures for approaching and recruiting eligible participants for MITS. Phenomenology was used in order to capture first-hand experiences from participants, providing a rich data to understand the experiences of parents and caretakers and the meaning of these experiences. To ensure trustworthiness, different methods and data collection instruments were used. In addition, different people worked on the data coding, and quality control was performed before and during the data analysis.

These results may be specific to the context of Hospital Central de Quelimane and therefore may not be generalizable to other settings where parents’ experiences may vary and be completely different. Only three families who refused consent for MITS were included in this study, which limited the exploration of the experience of this particularly relevant group of individuals, as most of the participants approached for MITS accepted participation. The team was involved through the observations, albeit not participant, in the process of the MITS consent request, which may have created some desirability bias.

### Conclusions

Participants’ consent to MITS was driven by the desire to learn about the CoD of their deceased child. The study identified that the experiences of parents and caretakers of deceased children were characterized by logistical and operational issues linked to the procedure itself (i.e. timing for performing MITS, seepage) and by personal, social and cultural issues (i.e. state of mind after the death, complex decision making processes within the family, washing of the body after a seepage for purification).

We recommend that when requesting consent for MITS, clear and understandable information is delivered. In places where post-mortem procedures are planned or are taking place, it is also necessary education campaigns to inform the community. Empathy should also be observed by healthcare providers asking for consent.

Furthermore, a profound knowledge of the social dynamics and cultural aspects around life and death is crucial in studies involving post mortem procedures.

## Supporting information

S1 Appendix Participant observation guide. (DOCX)

S2 Appendix Semi-structured interview guide for relatives of deceased children who accepted the MITS. (DOCX)

S3 Appendix Semi-structured interview guide for relatives of 3deceased children who refused the MITS. (DOCX)

S4 Appendix NVivo codebook. (DOCX)
