# Supplementary material for: Experiences of parents and caretakers going through the consent process to perform minimally invasive tissue sampling (MITS) on their deceased children in Quelimane, Mozambique: A qualitative study
Source: PLoS One. 2023 Jun 9;18(6):e0286785. doi: 10.1371/journal.pone.0286785 (PMC10256146; doi:10.1371/journal.pone.0286785)
Supplement: S1 Appendix — (DOCX) [file pone.0286785.s001.docx]

| **SSP_CHAMPS_001_A01_v02_PT** | | **Participant Observation**  **Death-related procedures in health facilities** | | |
| --- | --- | --- | --- | --- |
|  | ***Centro de Investigação em Saúde da Manhiça (CISM)***  **CHAMPS SBS**  **GUIÃO DE OBSERVAÇÃO PARTICIPANTE** | |  |  |

**1. INSTRUCTION**

1.1 This participant observation is to explore procedures related to death in Quelimane Central Hospital and other health facilities. The purpose is to observe health care providers during the execution of their daily activities in order to determine the procedures acceptable to them and the most appropriate strategies and approaches for future implementation of the MIA technique.

1.2. Go to the health facility to be observed and contact the health worker/s to be observed. Explain the CHAMPS program to him/her and ask permission to accompany him/her in his/her daily work.

1.3 In cases where the health care provider is dealing with relatives of the deceased or other colleagues, introduce yourself and ask permission to observe what is happening. If you feel it is appropriate, briefly explain the program.

1.4. concentrate on observing the themes defined below in the topic tab. Also observe the physical space, the relevant objects that are used and the time (sequences, duration of procedures and interactions between people).

1.5. Before you leave, you should inform the health professional/s that you have finished your work and thank him/her for being there. If you need to come back, set a date and time or ask if you can come back at any time.

1.6. Once in the office, record all the data you have obtained. No more than 2 days should pass between the observation and the registration, so as not to lose information. You must make a report of the observation following the point-by-point guide.

**Tips to remember what was observed:**

- Try to remember by making a historical sequence of events.
- Draw a map of the physical space where the observation took place (a room, an office, a corridor...). "Move" around the map, trying to remember the details of activities and conversations that took place in each place.
- Start with points (butllet points) and then develop each point in depth.

**2**. **GUIDE OF TOPICS/INFORMATION TO COLLECT**

**Site:** MOZ

**Observation ID:**

**Event type**: Death-related procedures in health facilities

**Place(s):**

**Date:**

Gatekeeper / Health personnel from whom permission was asked:

People with whom the observer interacted throughout the observation:

**Observer's name (initials):**

**Time of arrival of the observer:**

**Observer's departure time:**

**Introduction**

[Brief summary of what was observed and noteworthy themes. No more than one paragraph, or 10-15 lines] 2.4.

**Description of place(s)**

**Report the events in a chronological manner, taking into account:**

1. Activities, procedures, and circuits observed
2. Relationship and communication between health professionals and family members or clients

- How do health professionals approach family members?
- Describe whether health professionals ask for consent or explain procedures (anything) to clients; describe attitudes and manner of speaking of the health professional (whether he/she shouts, speaks softly, has to repeat the same thing many times, etc.);
- Describe reactions of users, family members, to explanations by health professionals (what words, expressions, gestures do they use?)

Describe communication between family members

1. Cases of occurrence of death during the observation

**1. Circuit of communication of death**

- Was the family present at the time of death? How did they react?
- How is the notification/confirmation of death done by the health professional (words, attitudes)? Who notifies/communicates? Who is present? To whom is it specifically addressed?
- How long after the occurrence of the death is there communication among family members?
- Reactions (crying, what is said, comments, etc.)
- Communication of death between family members (What means are used - physical meeting, phone call, SMS, message passed through third party)?
- Bureaucracy and documents that the hospital delivers to the family
- Location of the body during communication about the occurrence to the family members

2. Treatment of the body

- Is the body taken to the morgue?
- Is it washed at the hospital Who washes, material used, who gives material to wash the body, etc.

3. Transportation of the body

- How is it taken from the place of death to the morgue?
- How is it transported to the home? Who transports, conditions under which it is transported

D. Supports/incentives

1. Observe/Describe:

- Whether users comment on supports;
- If they demand/request any support, what kind of support (to whom requests are directed);
- Reaction of health professionals to requests for support.
- If health professionals make requests to family members/users, how do they do it?

E. Times

- Observe how long procedures take (medical exams, body wash, analysis, people involved, etc.)
- User reactions on waiting time (comments, attitudes, behaviors, etc.)

F. Description of persons observed:

- Complete description of all persons observed: apparent age, how they were dressed, role in the entire observation, relationship to the deceased (if applicable) and to the other persons present, decision makers.

G. Other issues to observe/capture

- Comments about deaths and burials among users;
- Comments about illnesses in general;

**Are there any issues that should be brought to the attention of other CHAMPS and mortality surveillance teams (Clinical CE, Demographics, COMSA...)?**
