# Supplementary material for: Experiences of parents and caretakers going through the consent process to perform minimally invasive tissue sampling (MITS) on their deceased children in Quelimane, Mozambique: A qualitative study
Source: PLoS One. 2023 Jun 9;18(6):e0286785. doi: 10.1371/journal.pone.0286785 (PMC10256146; doi:10.1371/journal.pone.0286785)
Supplement: S2 Appendix — (DOCX) [file pone.0286785.s002.docx]

**Instructions**

| 1. explain to the interviewee that:  - The purpose of this interview is to talk to the participants about the CHAMPS program (study) and aspects related to taking tissue and fluid samples from the body of recently deceased children in a minimally invasive manner (MITS).  - You have been invited for this interview because due to your recent experience with CHAMPS and MITS, after the death of your child, your contribution is essential to understand the above topics from the family's perspective.  - We will start our conversation with issues related to informed consent. Then we will continue with aspects related to taking tissue and fluid samples from the body of recently deceased children. Finally we will talk about the results derived from the analysis of these tissue and fluid samples and about their delivery to the families of the deceased children. The interview is expected to last 40 minutes- 1 hour.  2. Say that, if you allow, the interview will be recorded, just so that we do not run the risk of losing important information that you are going to give during the interview. If you do not agree to have it recorded, the interviewer will take notes as the conversation goes on.  3. Also say that although the interview will be recorded, you will also take notes throughout the conversation to ensure the security of the information. All recorded information will be confidential and you will not be identified by name. |
| --- |

1. **DEMOGRAPHIC INFORMATION**

Audio File Ref / Study Number

CHAMPS_MZ_SSI_FM_|__|__| - |__|__|__|__|__|

(ID file) (Participant ID - No and 3 letters)

Participant Data Gender |__| Age |__|| Born in (province and town): _____________________ ________________________________________________________________________________________

Living in (P. Admin. and neighborhood): _______________________________________________________________

Education level (last completed level): ____________________________________________________

Occupation: _______________________________________________________________________________

Religion and church: __________________________________________________________________________

Category of the respondent in relation to the deceased (e.g. mother/father, uncle, grandfather/grandmother, brother/sister...): _____________________________________________________

Data of the deceased

Gender|__| Age |__|__| ______________

(days/months/years)

Date of death |__|__|/|__|/|__||__|| Stillbirth 0 Stillbirth 0 Abortion 0

Date and place of interview |__|||/|__||/|__|||| _________________________________________________________

Línguas faladas ________________________________________________________________________________________

Interview result

Recorded 0 Not recorded 0 Reason:____________________

___________________________________________________________

Complete 0 Interrupted 0 Reason: __________________

___________________________________________________________ Unable to complete 0

To be completed on (date):

_________________________

Interviewer: |__|__|__|MITS

| 1. Can you explain to me how the child died?  [Briefly. This is about knowing how the child died, where they sought treatment, and what happened at the health facility]  2. Can you explain to me how the request for consent to perform MITS on the deceased child was carried out?  3. What do you think about the request for consent that was made to perform MITS on the deceased child? Why?  Explore what the interviewee thinks about:  The manner in which the request for consent was carried out?  The time and place when consent was asked/requested?  The time it takes to ask for consent?  The person asking or requesting consent?  Whether the request was made to the right person?  The information conveyed?  The best way to ask for consent?  4. How was the decision to perform MITS reached?  Explore:   - Reasons for accepting MITS? - People involved? - People who had the final say? - Difficulties in making the decision?  1. What do you think about the MITS that was performed on the deceased child's body? 2. Explore what the interviewee thinks about: 3. The length of time MITS was performed? 4. Place where MITS was performed? 5. The health care professionals involved? 6. The condition in which the body was returned? 7. Changes that MITS caused in the program for conducting the funeral? 8. What was talked about in informed consent, is that what happened? 9. Do you think someone should have accompanied the performance of MITS? If yes, who? Why? 10. Did the fact that you performed MITS, affect the emotions, feelings of the family? 11. And did it affect the cost of the ceremonies and program for holding the funeral for the deceased child? How? 12. How would you feel if your home or another place in your community had been used as a place to do MITS on the body of the deceased child?   Explore:  Barriers and difficulties?.  How could the CHAMPS program improve? | **RESUMO** |
| --- | --- |

1. **DELIVERY OF MITS RESULTS**

| 1. What do you think about the delivery of results derived from MITS that was done to deceased child?  Explore :  Whether or not the participant received the results. If not, explore whether the team has contacted him/her and has information on when and how the results will be delivered.  If the results were delivered, explore how the participant feels about:  The amount of time it took to wait or that he/she is expecting the results?  How you were contacted and informed?  People involved (who gives and who receives the results)  Language used?  Are there any issues that came up that should be brought to the attention of other CHAMPS teams (EC, Clinical, Demographics...)?  ____________________________________________________________________________________________________________________________________________________________________________________________________________________________________________________________________________________________________________________________________________________________________________________________________________________________________________________________________________________________________________________________________________________________________________________________________________________________________________________________________________________________________________________________________________  COMMENTS: | **RESUMO** |
| --- | --- |

**Há algum tema que surgiu que deva se pôr em conhecimento de outras equipas CHAMPS (CE, Clínica, Demografia...)?**

________________________________________________________________________________________________________________________________________________________________________________________________________________________________________________________________________________________________________________________________________________________________________________________________________________________________________________________________________________________________________________________________________________________________________________________________________________________________________________________________________________________________________________________________________________

**OBSERVAÇÕES:**

________________________________________________________________________________________________________________________________________________________________________________________________________________________________________________________________________________________________________________________________________________________________________________________________________________________________________________________________________________________________________________________________________________________________________________________________________________________________________________________________________________________________________________________________________________________________________________________________________________________________________________________________________________________________________________________________________________________________________________________________________________________________________________________________________________________________________________________________________________________________________________________________________________________________________________________________________________________________________________________________________________________________________________________________________________________________________________________________________________________________________________________________________________________________________________________________________________________________________________________________________________________________________________________________________________________________________________________________________________________________________________________________________________________________________________________________________________________________________________________________________________________________________________________________________________________________________________________________________________________________________________________________________________________________________________________________________________________________________________________________________________________________________________________________________________________________________________________________________________________________________________________________________________________________________________________________________________________________________________________________________________________________________________________________________________________________________________________________________________________________________________________________________________________________________________________________________________________________________________________________________________________________________________________________________________________________________________________________________________________________________________________________________________________________________________________________________________________________________________________________________________________________________________________________________________________________________________________________________________________________________________________________________________________________________________________________________________________________________________________________________________________________________________________________________________
