# Supplementary material for: Experiences of parents and caretakers going through the consent process to perform minimally invasive tissue sampling (MITS) on their deceased children in Quelimane, Mozambique: A qualitative study
Source: PLoS One. 2023 Jun 9;18(6):e0286785. doi: 10.1371/journal.pone.0286785 (PMC10256146; doi:10.1371/journal.pone.0286785)
Supplement: S4 Appendix — (DOCX) [file pone.0286785.s004.docx]

SBS COMSA

Nodes

| **Name** | **Description** |
| --- | --- |
| General experience | General information about parents' experiences of death and postmortem procedures. |
| Post-MITS experience | All General Information on Parents' Experiences of Death and Postmortem Procedures |
| Family Member's Participation in MITS | Considerations on the participation of the child's relatives in the timing of the MITS |
| Perception of MITS completion time | All related to the timing of MITS |
| Translocation of deceased children's bodies after MITS | All aspects related to the post-MITS transfer of children's bodies to the community |
| Perceptions on consent for MITS | All aspects of perception about consent to MITS |
| Experiences of consent after child death | All aspects related to participants' experiences with MITS consent |
| Perception of susceptibility |  |
| Perception about MITS | All aspects related to perceptions about MITS |
| Negative Experiences post MITS | All aspects related to parents' and relatives' experiences about negative experiences after MITS |
| Positive Experiences post MITS | All aspects related to parents' and family members' experiences about positive experiences after MITS |
| Time to complete MITS | Perceptions about the time needed to perform MITS |
| Perception of the benefits of MITS | All events related to child deaths in the community |
| Recommendations |  |
| Decision to undertake MITS | All aspects related to decision making in families |
| Who makes the decision to do MITS | All information related to the decision-maker for accepting MITS |
| Who makes the decision to decline MITS | All information related to decision-maker for denial of MITS |
| Reasons for accepting MITS | All aspects related to the reasons given by parents or relatives of the deceased child for accepting MITS |
| Reasons for declining MITS | All aspects related to the reasons given by the parents or relatives of the deceased child for refusing MITS |
